# Supplementary material for: Global landscape of replicative DNA polymerase usage in the human genome
Source: Nat Commun. 2022 Nov 24;13:7221. doi: 10.1038/s41467-022-34929-8 (PMC9700718; doi:10.1038/s41467-022-34929-8)
Supplement: Supplementary file 1 — Supplementary Information [file 41467_2022_34929_MOESM1_ESM.pdf]

SUPPLEMENTARY INFORMATION

Global landscape of replicative DNA polymerase usage in the human genome

Eri Koyanagi[1,#], Yoko Kakimoto[1,#], Tamiko Minamisawa[2,#], Fumiya Yoshifuji[3,#], Toyoaki Natsume[4,5,†], Atsushi Higashitani[3], Tomoo Ogi[6], Antony M. Carr[7], Masato T. Kanemaki[4,5,8], Yasukazu Daigaku[1,2]*

[1]Frontier Research Institute for Interdisciplinary Sciences, Tohoku University, Sendai, Japan
[2]Cancer Genome Dynamics project, Cancer Institute, Japanese Foundation for Cancer Research, Tokyo, Japan
[3]Graduate School of Life Sciences, Tohoku University, Sendai, Japan
[4]National Institute of Genetics, Research Organization of Information and Systems (ROIS), Mishima, Japan
[5]Department of Genetics, The Graduate University for Advanced Studies (SOKENDAI), Mishima, Japan
[6]Research Institute of Environmental Medicine, Nagoya University, Nagoya, Japan
[7]Genome Damage and Stability Centre, School of Life Sciences, University of Sussex, Falmer, United Kingdom, BN1 9RQ
[8]Department of Biological Sciences, Graduate School of Science, The University of Tokyo, Tokyo, Japan

[#]These authors contributed equally to this work.

[†]Present address: Research Center for Genome & Medical Sciences, Tokyo Metropolitan Institute of Medical Science, Tokyo, Japan

*Corresponding author (e-mail: yasukazu.daigaku@jfcr.or.jp)

Supplementary Fig. 1

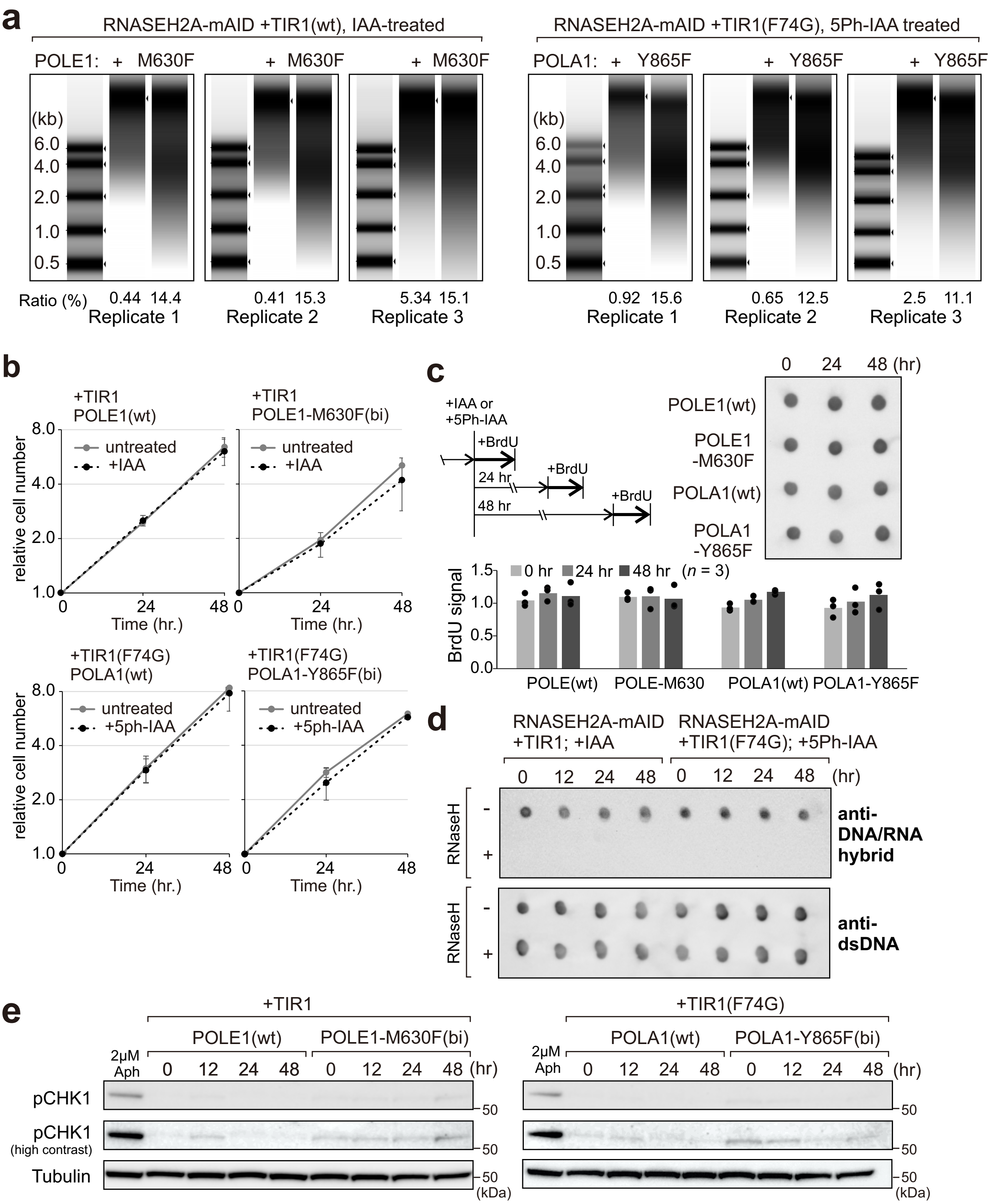

Supplementary Fig. 1 **The condition of cells following the initiation of RNASEH2A degradation.**

a Extracted genomic DNA from the indicated cell lines after the initiation of RNASEH2A degradation was treated with alkali to cleave at incorporated rNMPs and analysed by electrophoresis. These DNA samples are used for replicates of Pu-seq experiments. 'Ratio' indicates the percentage of DNA < 2.0 kb. **b** The relative cell number 24, 48 hr after the addition of IAA to +TIR1 cells or 5ph-IAA to +TIR1(F47G) cells (0 hr = 1.0). Data represents the mean **±** s.d of *n* = 3 independent experiments. **c** Detection of BrdU incorporation into genomic DNA at 0, 24, 48 hr following the addition of IAA to +TIR1 cells or 5ph-IAA to +TIR1(F47G) cells. Top left: the experimental scheme. Cell were pulse-labelled for 2 hours with 10 μM of BrdU at the 0, 24, 48 hr time points. Top right: immunostaining of BrdU incorporated in genomic DNA. 800 ng of DNA was spotted in each dot. Bottom: quantification of BrdU signals from *n* = 3 indepedent experiments. Data represents the mean (bar) and individual values (circle). **d** Dot blot assay to detect DNA/RNA hybrid structures. Extracted genomic DNA was treated with RNaseA only (RNaseH -) or RNaseA and RNaseH (RNaseH +) and applied to dot blot to perform immunostaining with the S9.6 antibody, which recognises the DNA/RNA hybrid structures, and anti-dsDNA antibody. **e** Chk1 phosphorylation at 0, 12, 24, 48 hr following the addition of IAA to +TIR1 cells or 5ph-IAA to +TIR1(F47G) cells. Source data of a, b, c, d and e are provided as a Source Data file.

Supplementary Fig. 2

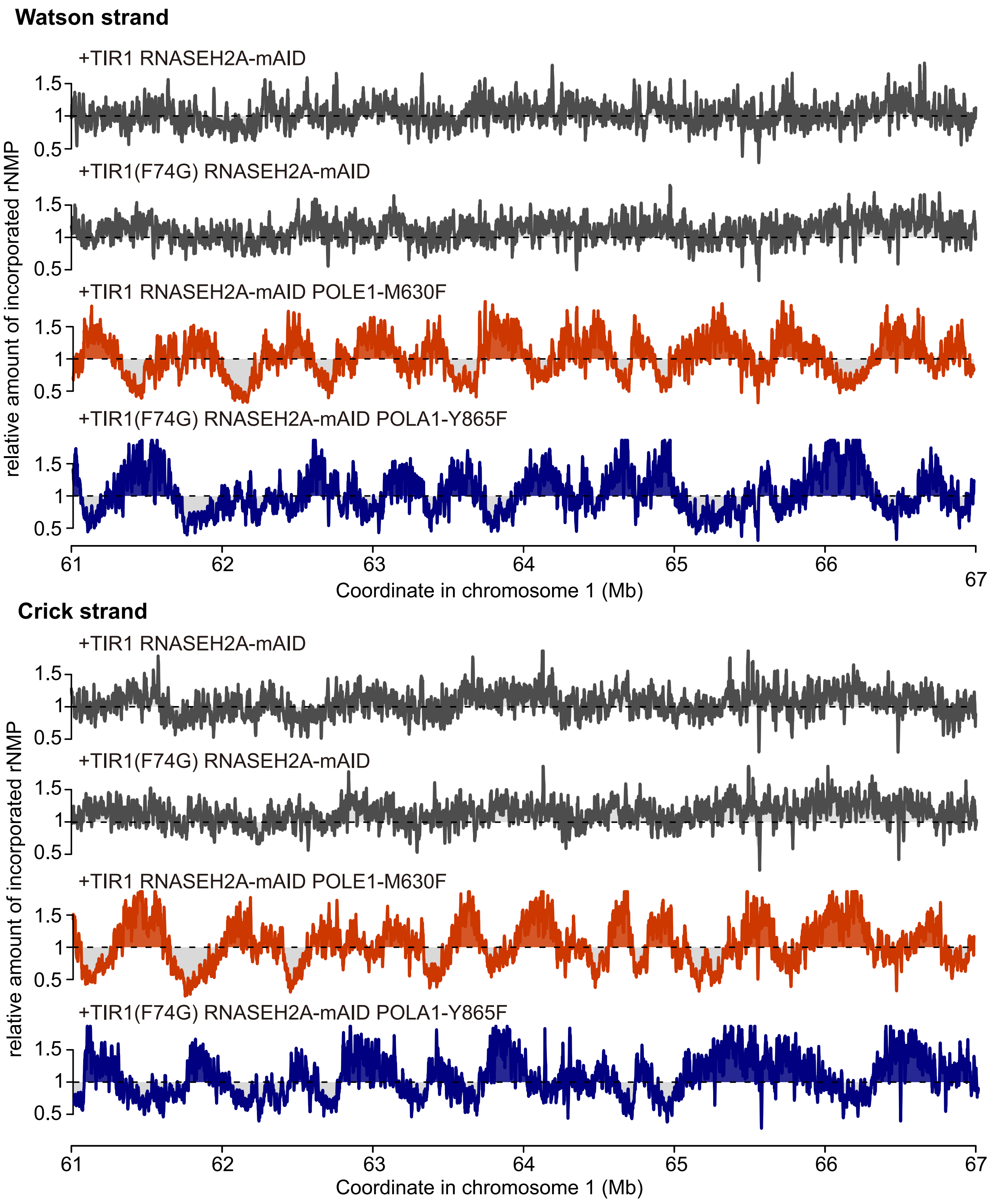

Supplementary Fig. 2 **Relative scores of rNMP incorporation in POLE1-M630F, POLA1-Y865F and POL+ with RNaseH2 degradation.** The relative read number on the Watson and Crick strand for each 1kb bin are shown (1 = genomic average). Gray: Pol^{+}, Orange: Polε (POLE1-M630F). Blue: Polα (POLA1-Y865F). Data were smoothed with a moving average (m = 3; see materials and methods).

Supplementary Fig. 3

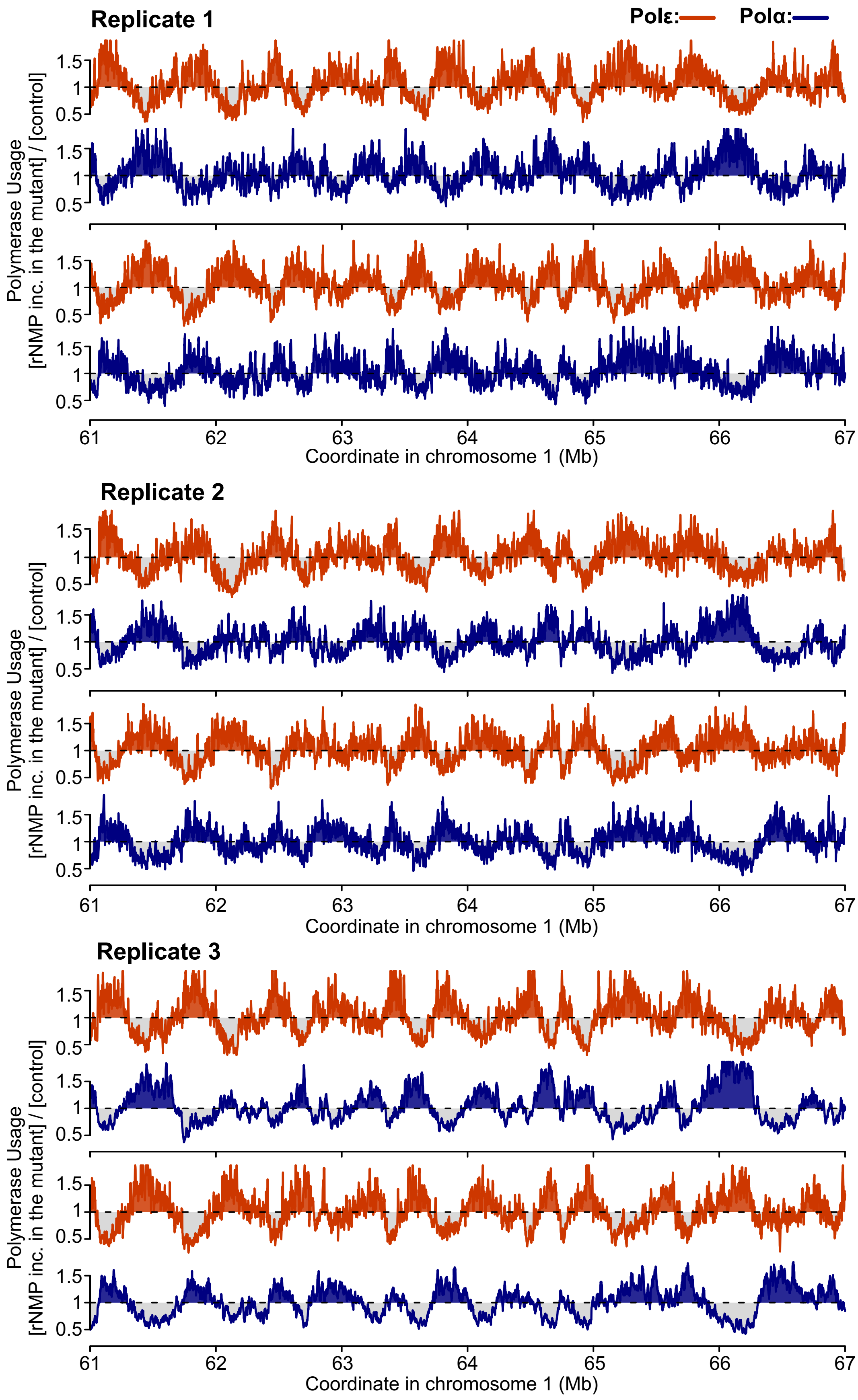

Supplementary Fig. 3 **Polymerase usage of 3 experimental replicates across the human genome.** For three independent experiments (Replicate 1, 2 and 3) the profiles of the relative reads for Polε and Polα mutants on the Watson and Crick strand for each 1kb bin are shown. Data are normalised to Pol+. Orange: Polε (POLE1-M630F). Blue: Polα (POLA1-Y865F). A representative region of chromosome 1, a different location to that in Fig. 2, is shown. Data were smoothed with a moving average (*m* = 3; see materials and methods).

Supplementary Fig. 4

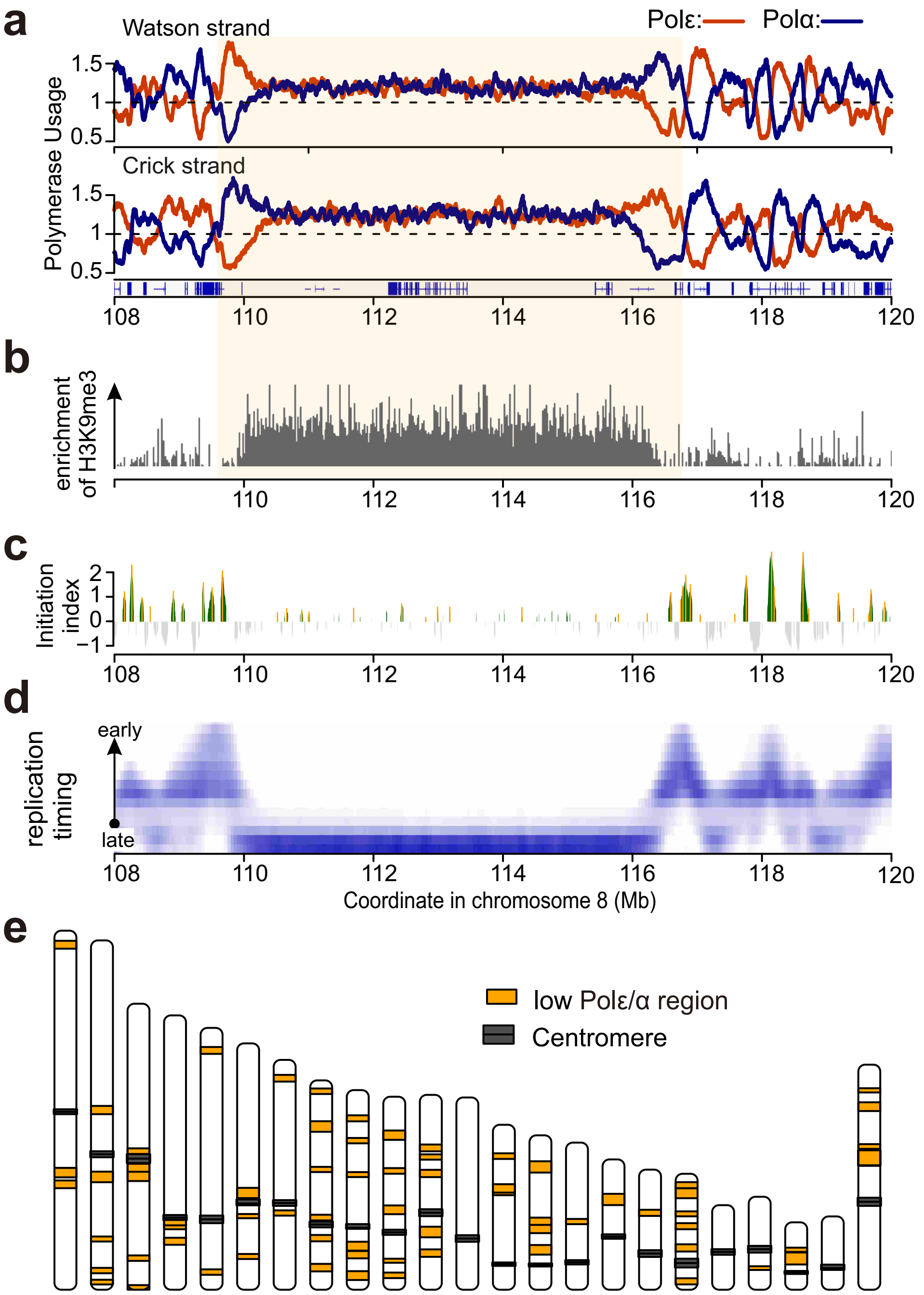

Supplementary Fig. 4 **Polymerase usage and replication initiation around late replicating regions.** **a** Polymerase usage profiles of Polε (orange) and Polα (blue) plotted for a representative region showing an equal profile of polymerase usage. Light orange background represents such a 'low Polε/α region'. **b** H3K9me3 enrichment at the same region. Data are taken from Lay et al (2015)[1]. H3K9me3 marks heterochromatin. **c** Initiation index plotted for the same region. **d** Replication timing (RT) in HCT116 cells. RT data are taken from Zao et al (2020)[2]. **e** Schematic of the genome-wide distribution of 'low Polε/α regions'.

Supplementary Fig. 5

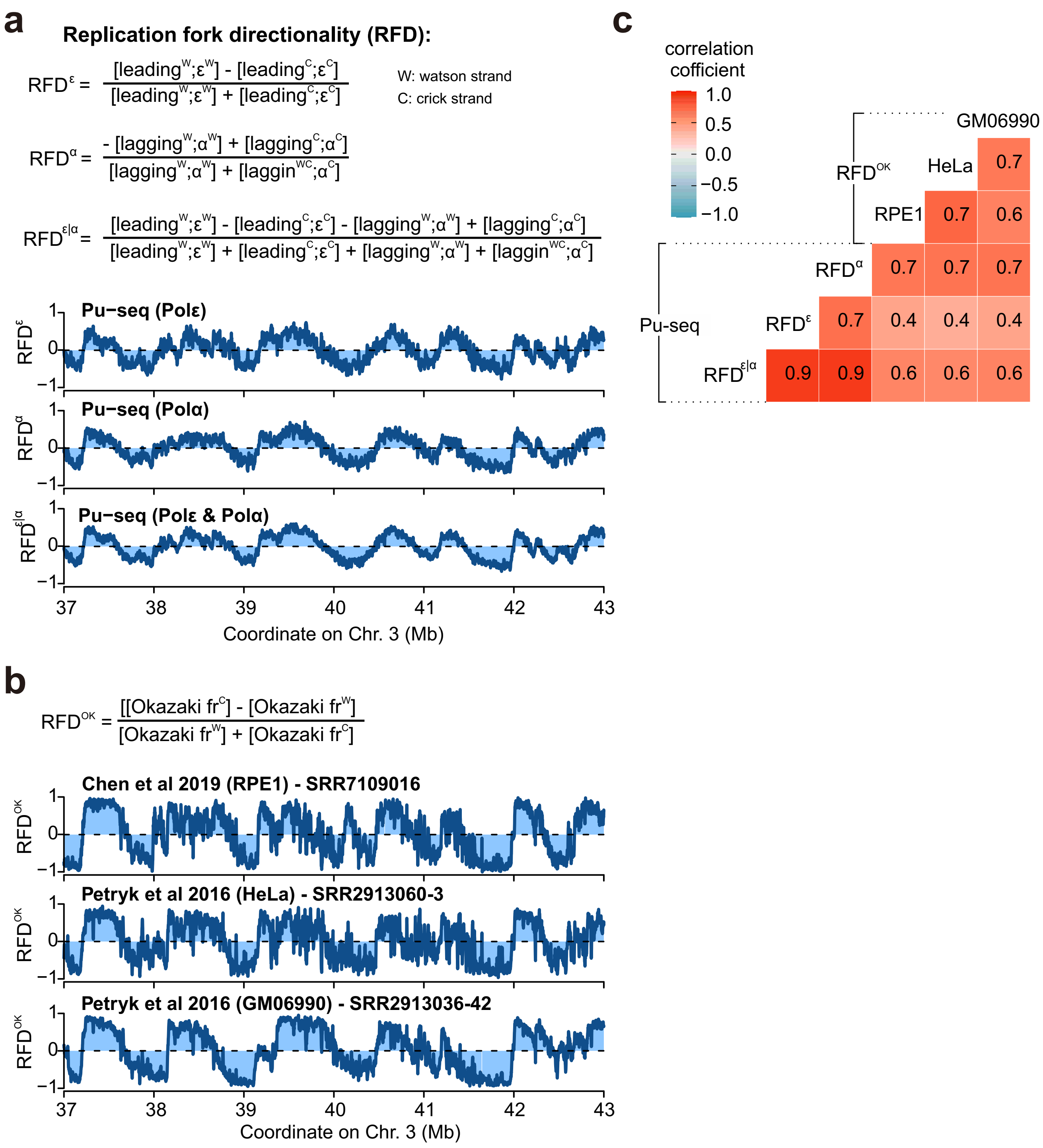

Supplementary Fig. 5 **Replication fork directionality (RFD) from Pu-seq and OK-seq.**
a RFDs are calculated from Polε usage (RFD^{ε}), Polα usage (RFD^{α}) or both data sets ($RFD^{\varepsilon|\alpha}$) and plotted for a representative section of chromosome 3. **b** RFDs are calculated from three published OK-seq datasets and plotted for the same representative region. **c** Correlation among RFDs from Pu-seq and OK-seq.

Supplementary Fig. 6

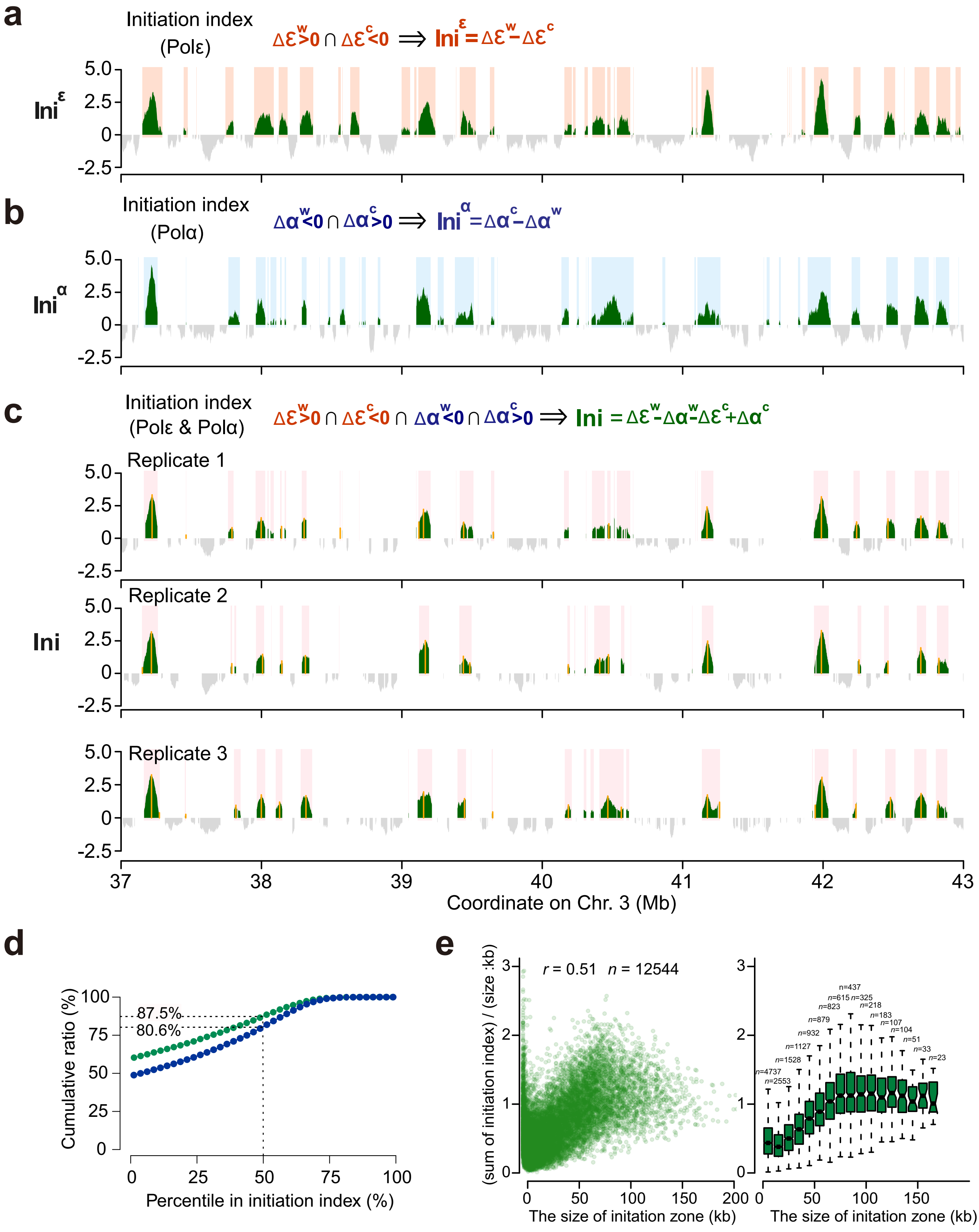

Supplementary Fig. 6 **Genome-wide initiation index identifies replication initiation zones.**

a Initiation index calculated from Polε and plotted for a representative region of chromosome 3. Replication initiation sites are defined as the positive zones of initiation index and are marked with a coloured background. **b** Initiation index calculated from Polα data and plotted as in panel a. **c** Initiation index calculated from combined Polε and Polα data and plotted as in panel a. The three independent experimental replicates are shown. Yellow vertical lines indicate computationally identified location of highest points within the zone. **d** Cumulative ratio of replication initiation sites which locate at the concordant regions between the two experimental replicates (green: replicate 1 & 2) and among three replicates (blue). **e** The relationship of initiation index per 1 kb with the width of initiation zones (*n* = 12544 initiation zones). *r* represents Pearson correlation coefficient. For the boxplots, the horizontal black lines within boxes represent median values, boxes indicate the upper and lower quartiles (25-75%), and whiskers indicate the 1.5× interquartile range.

Supplementary Fig. 7

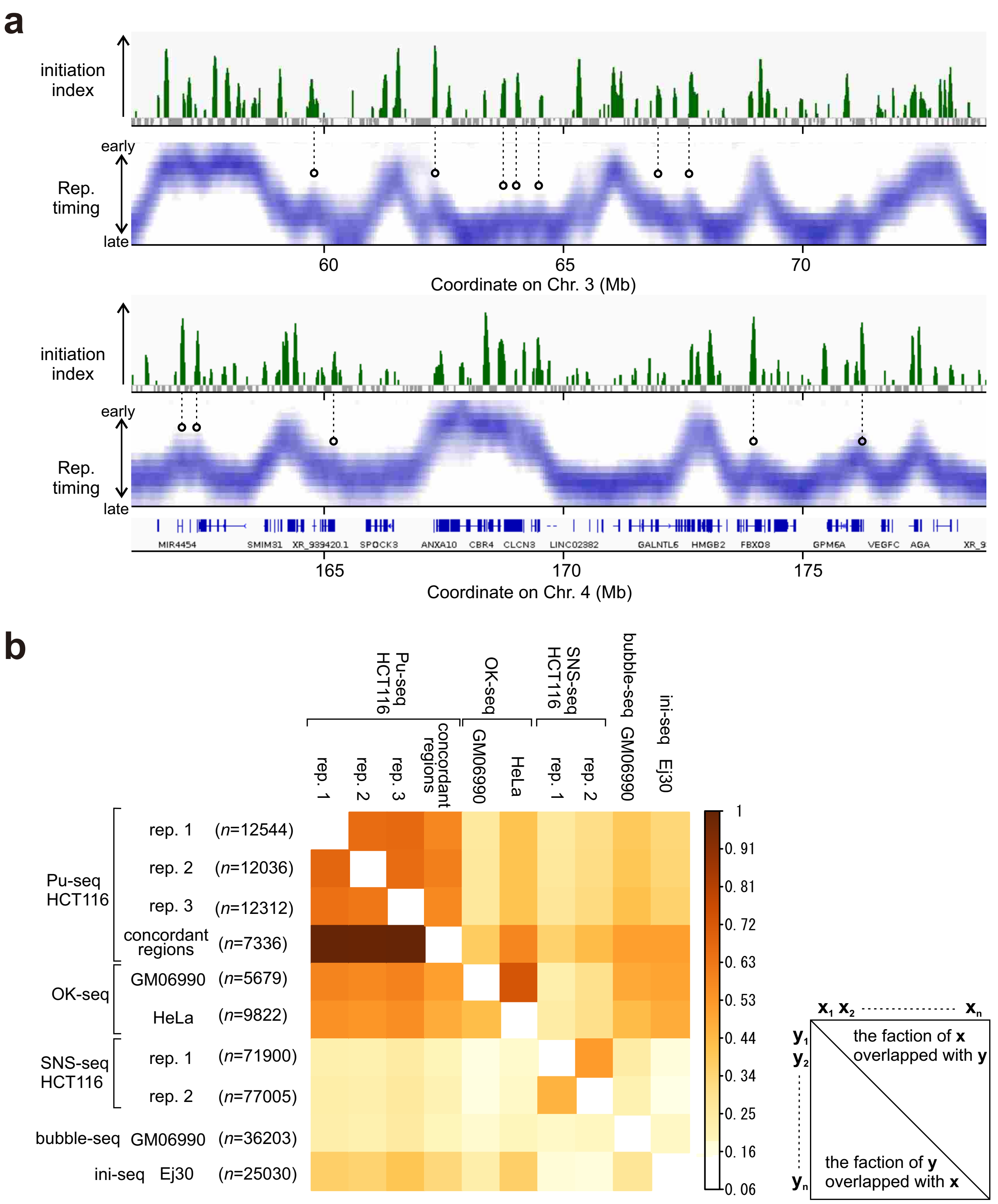

Supplementary Fig. 7 **Genome-wide initiation index compared to replication timing and various techniques that detect replication initiation zones.**

a High-resolution replication timing data and initiation index plotted for a representative region of chromosome 3 (top) and chromosome 4 (bottom). The profile of high resolution replication timing for HCT116 cells are from Zao et al (2020)[2]. **b** Comparison of initiation zones detected by various techniques. The heatmap represents the intersection fraction of pair-wise overlap for initiation sites. Data derived from OK-seq were obtained from the GitHub page of Chun-Long Chen (https://github.com/CL-CHEN-Lab/OK-Seq). Data of initiation site SNS-seq, bubble-seq and ini-seq are derived from Thakur et al (2022)[3], Mesner et al (2013)[4] and Langley et al (2016)[5]. Analysis was performed using the 'intervene' tool[6] (*n*: the number of initiation sites)

Supplementary Fig. 8

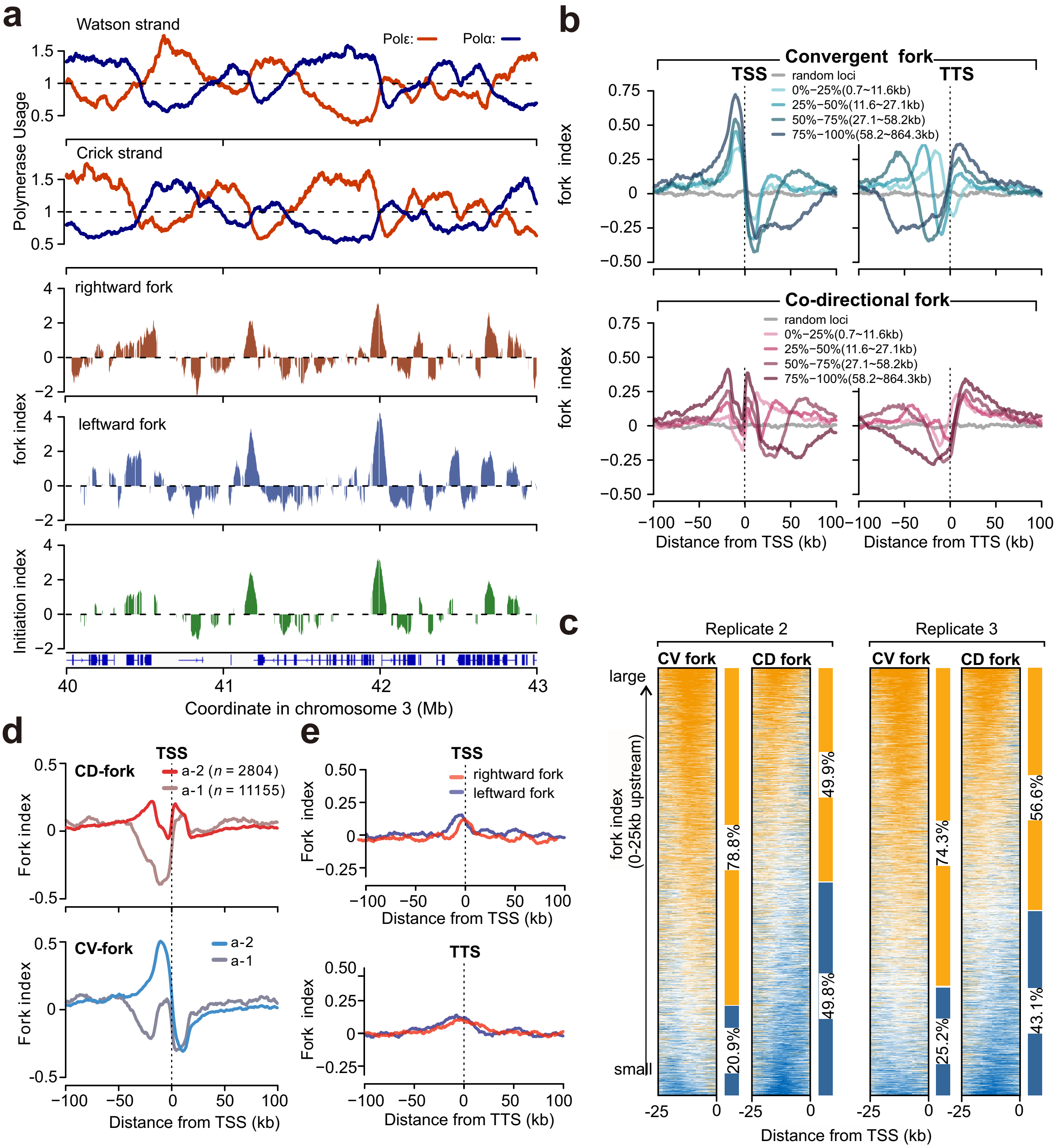

Supplementary Fig. 8 **The profiles of rightward and leftward forks.**

a The fork index profile of an experimental replicate. Top: Profiles of polymerase usage plotted for a representative region of chromosome 3. Middle: leftward and rightward moving fork index (Fk^R and Fk^L, see Fig. 5a) plotted for the same region. Bottom: initiation index plotted for the same region. Data used in these plots are derived from the experimental replicate 2 and are comparable to those from the experimental replicate 1, which is shown in Fig. 5b. **b** Averaged fork index +/- 100 kb around annotated TSS and TTS. Data for the fork indices of CV and CD forks are categorised by gene length. Only the 50% most transcriptionally active genes were included. **c** Heat map representation of CV and CD fork index at 25-0 kb upstream regions of active TSS. Equivalent data for replicate 1 are shown in Fig. 5e. **d** Fork index around TSS (+/- 100 kb) of active genes. Data for the fork index around genes with > average transcriptional activity are categorised into two groups as shown in Fig. 4a; a-1: the sum of initiation index in the upstream of TSS is less than or equal to 0 (i.e. average value), and a-2: this value is greater than 0. **e**. Fork index around TSS and TTS of non-transcribed genes. The rightward (orange) and leftward (blue) fork index data are identical to those of CD and CV forks which are not affected by transcription ('0%': grey lines) in Fig. 5d.

Supplementary Fig. 9

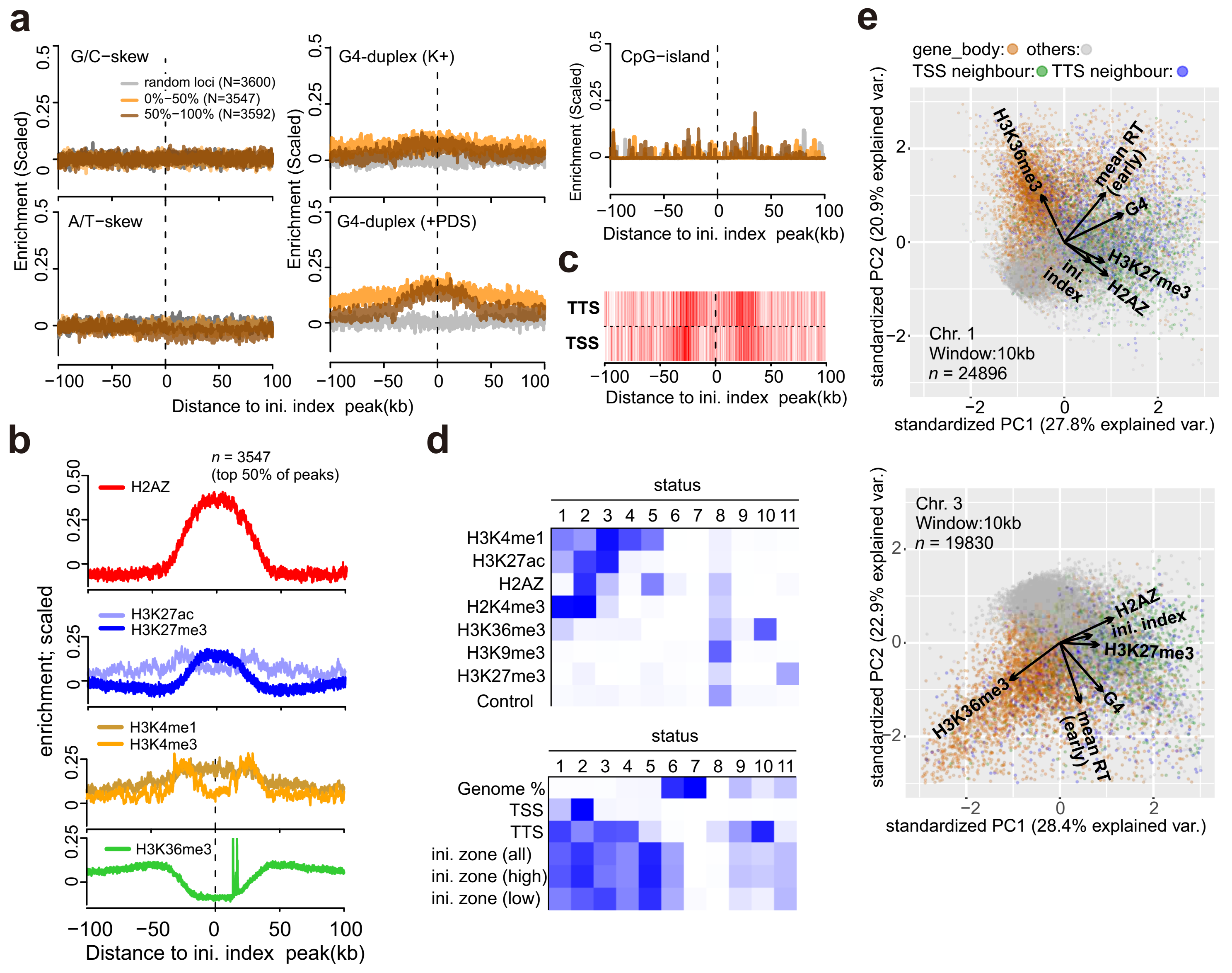

Supplementary Fig. 9 **Sequence features and chromatin status at replication initiation zones.**

a Enrichment of sequence-based features G/C-skew, A/T-skew, G4-duplex and CpG-islands +/- 100 kb around the computationally detected peaks (yellow vertical lines in Supplementary Fig. 6d) in initiation zones that are concordantly detected in the two experimental replicates. Plotted data are categorised by height of initiation index peaks; low: 0%-50%, high: 50%-100%. Data for genome-wide G4-duplex formation are derived from G4-seq published in Marsico et al (2019)[7]. K+; G4 duplexes formed in the presence of potassium ions. +PDS; G4 duplex formed in the presence of G4-targeting small molecule pyridostatin (PDS). **b** Equivalent analysis for H2AZ, H3K27 acetylation (ac), H3K27 tri-methylation (me3), H3K4 mono-methylation (me1), H3K4me3 and H3K36me3. Histone modification data are derived from ChIP-seq data for HCT116 published in Lay et al (2019)[1] and these genome-wide data were z-score normalised before averaging around peaks of initiation index. **c** Distribution of TSS and TTS around peaks of initiation index. **d** Heat maps for the model of chromatin-state annotation produced by ChromHMM as described in Ernst and Kellis (2012)[8]. Top: the model was trained on the datasets indicated by the chromatin modification and defined 11 genomic status bins (status 1-11) that represent different chromatin states. The relative enrichments of chromatin features that are present in each status bin are represented by the extent of blue shading (for example, chromosome status bin 10 is enriched only for H3K36me3, which distinguishes it from the remaining 10 bins). Bottom: the model then allocates the locations associated with the TSS, TTS and initiation zones to the same 11 bins (status 1-11), again representing the relative % occupancy by shade of blue (for example, most TSS are associated with bin 2. Referring back to the top heatmap reveals the correlating chromatin marks). **e** Principal component analysis to generate a correlation map of initiation index scores with the indicated genomic features. Top: chromosome 1 (*n* = 24896 genomic locus). Bottom: chromosome 3 (*n* = 19839 genomic locus). The arrows approximate the direction of each feature and their lengths approximate variances of the data.

Supplementary Fig. 10

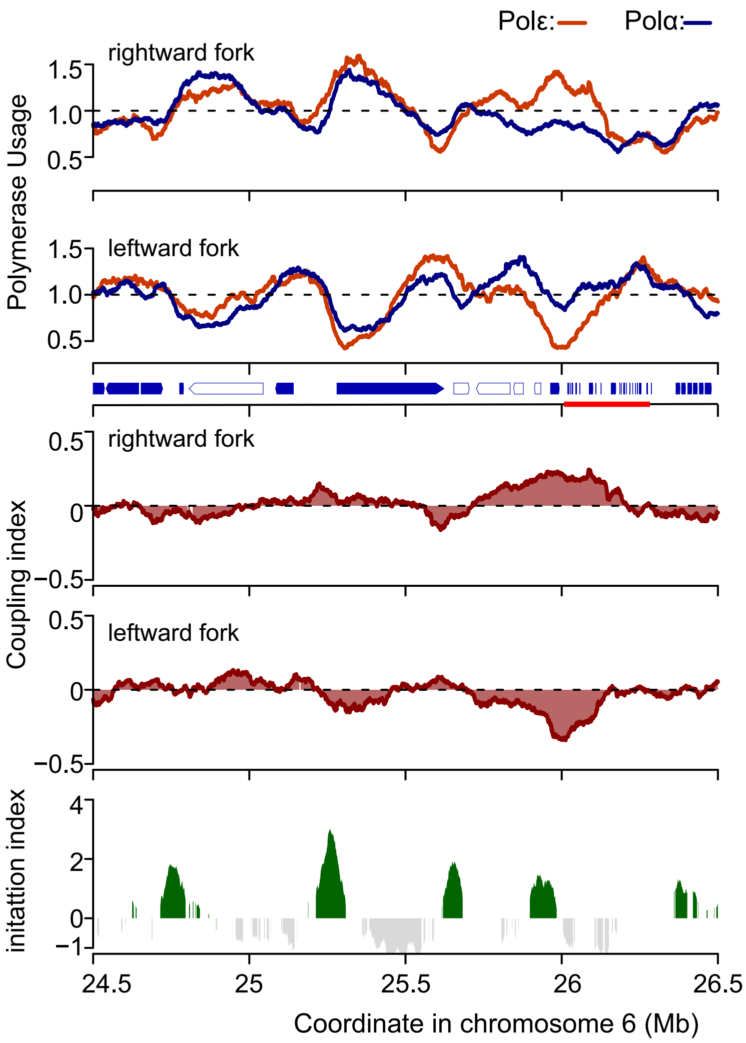

Supplementary Fig. 10 **DNA polymerase coupling/uncoupling in replicate 2.**
Equivalent data for replicate 2 as presented across the same representative region of chromosome 6 for replicate 1 in Fig. 6a,c. Top: Profiles of polymerase usage. Arrows on the horizontal axis indicate active genes (blue-filled) and inactive genes (unfilled). The red line on the horizontal axis indicates a cluster of histone-encoding genes. Middle: coupling index of rightward and leftward moving forks. Bottom: initiation index of the same region for comparison.

Supplementary Fig. 11

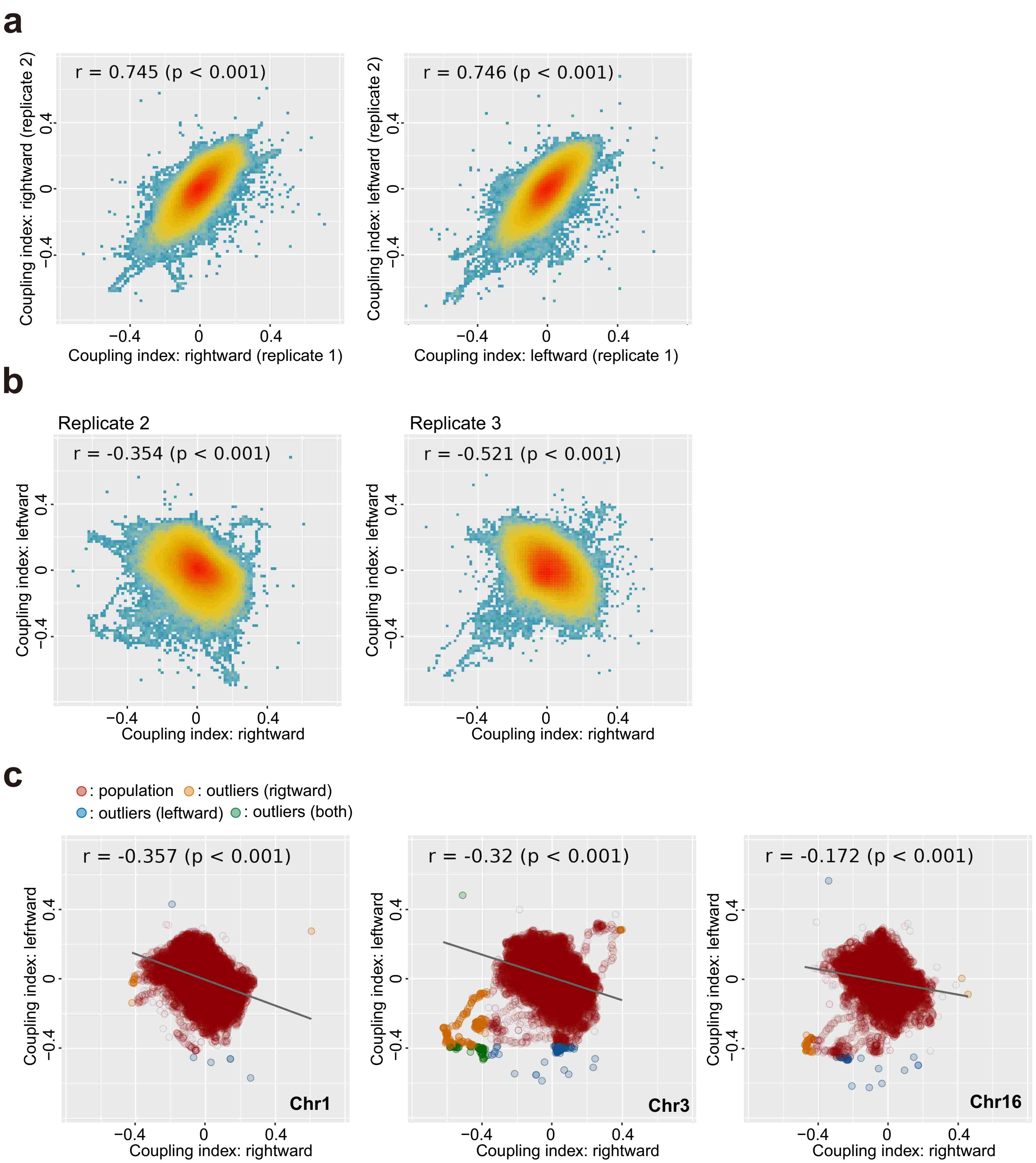

Supplementary Fig. 11 **The distribution of coupling index.**

a correlation of coupling index (CI, see Fig. 6b) derived from the experimental replicate 1 and 2. left: rightward moving forks, right: leftward moving forks. **b** Correlation of CI of rightward and leftward moving forks from replicate 2 and 3. See Fig. 6d for equivalent analysis for replicate 1. **c** Correlation of CI of rightward and leftward moving forks from replicate 2 presented by chromosome. Chromosomes 1, 3 and 16 are shown. Equivalent data for replicate 1 are shown in Fig. 7a. *r* represents Pearson correlation coefficient and *p* shows its statistical significance (one-tailed t-test).

Supplementary Fig. 12

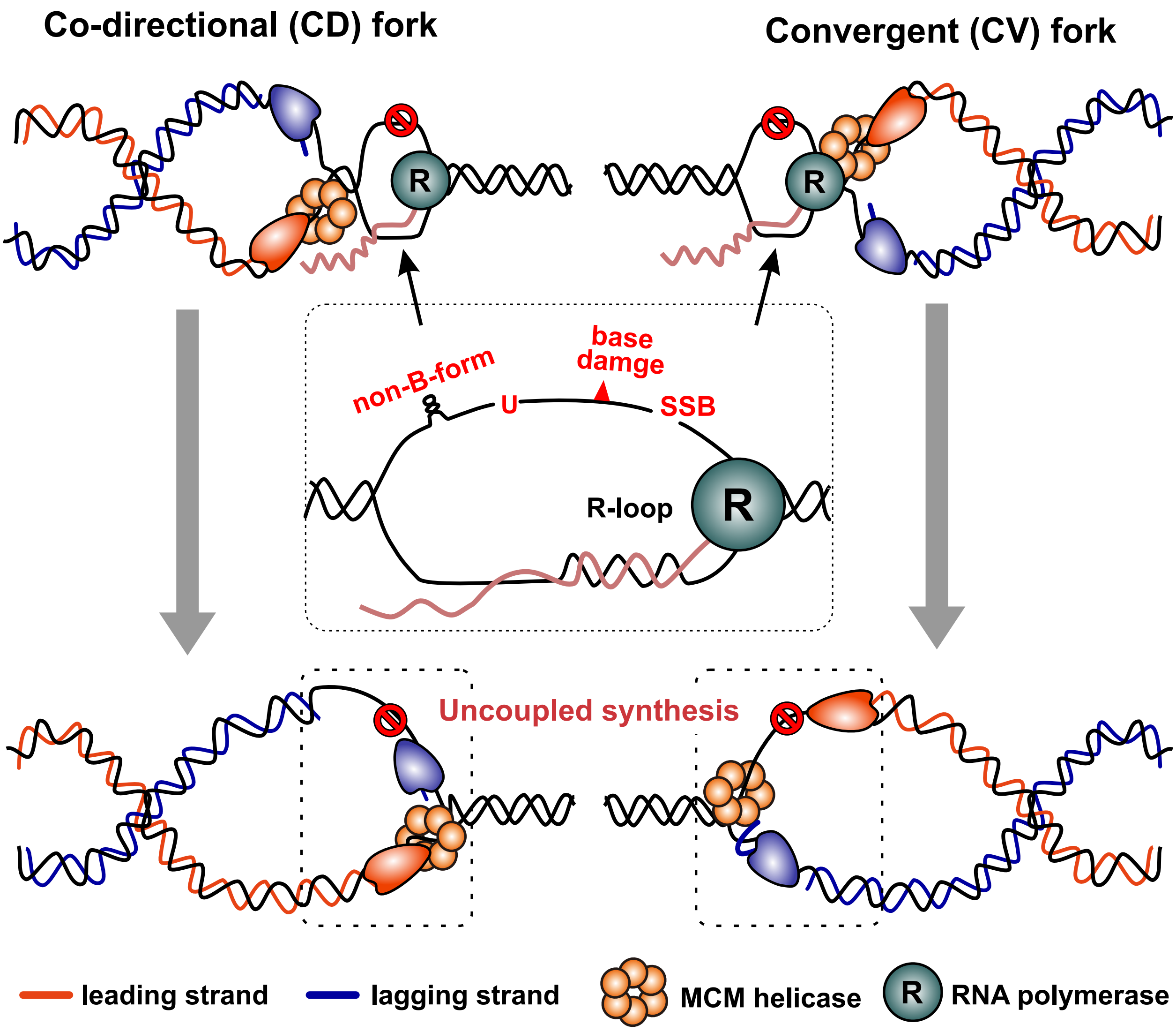

Supplementary Fig. 12 **The model of blocking DNA synthesis on the non-transcribed strand.** The R-loop formation exposes the opposite non-transcribed strand as ssDNA, which is susceptible to DNA damaging agents, DNA-modification enzymes (e.g. polynucleotide deaminase) or transformation to a non-B form DNA (e.g. G4-duplex). When codirectional or convergent forks proceed to the transcribed region, these replication blocking structures stall DNA synthesis on the non-transcribed strand.

Supplementary Table 1

Resource table

REAGENT	SOURCE	IDNTFIER
**Cell lines**		
HCT116 +TetOsTIR1(bi)	Natsume et al. 2016[9]	RIKEN NBRP RCB4662
HCT116 Tet-OsTIR1(bi)::AAVS1(bi) RNASEH2A-mAID-Clover::hygR(bi)	This study	
HCT116 Tet-OsTIR1(bi)::AAVS1(bi) RNASEH2A-mAID-Clover::hygR(bi) POLE1-L630F(bi)	This study	
HCT116 CMV-OsTIR1(F74G)::AAVS1(bi)	Yesbolatova et al. 2020[10]	
HCT116 CMV-OsTIR1(F74G)::AAVS1(bi) RNASEH2A-mAID-Clover(bi)	This study	
HCT116 CMV-OsTIR1(F74G)::AAVS1(bi) RNASEH2A-mAID-Clover(bi) POLA1-Y865F(bi)	This study	
**Oligo DNA**		
GATGAGATTAAGAGCAAGCTTGCCTCCCTGAAGGACGTTCCCAGCCGCATCGAGTGTCCACTCATCTACCACCTGGACGTGGGGGCC**t**T**c**TACCCCAACATCATCCTGACCAACCGCCTGCAGGTGA	IDT Alt-R™ HDR Donor Oligo, custom production	ssODN-POLE1-M630F
TTCTTTAGACTTTTTATGACGTGGCTTTTTAATTTCAGGTTTTTATGATAAGTTCATTTTGCTTCTGGACTTCAACAGTCTAT**tc**CCTTCCATCATTCAGGAATTTAACATTTGTTTTACAACAGTA	IDT Ultramer DNA Oligo, custom production	ssODN-POLA1-Y865F
**Oligo RNA**		
/AlTR1/**CAGGAUGAUGUUGGGGUACA**GUUUUAGAGCUAUGCU/AlTR2/	Alt-R CRISPR-Cas9 crRNA	crRNA-POLE1-M630F
/AlTR1/**UUAAAUUCCUGAAUGAUGGA**GUUUUAGAGCUAUGCU/AlTR2/	Alt-R CRISPR-Cas9 crRNA	crRNA-POLA1-Y865
**Plasmid DNA**		
px330-RNaseH2A-C-terminal	This study	
pBS-RNaseH2A -mAID-Clover-Hygro-donor	This study	

SUPPLEMENTARY REFERENCES

1. Lay FD, et al. The role of DNA methylation in directing the functional organization of the cancer epigenome. Genome Res 25, 467-477 (2015).

2. Zhao PA, Sasaki T, Gilbert DM. High-resolution Repli-Seq defines the temporal choreography of initiation, elongation and termination of replication in mammalian cells. Genome Biol 21, 76 (2020).

3. Thakur BL, et al. Convergence of SIRT1 and ATR signaling to modulate replication origin dormancy. Nucleic Acids Res 50, 5111-5128 (2022).

4. Mesner LD, Valsakumar V, Cieslik M, Pickin R, Hamlin JL, Bekiranov S. Bubble-seq analysis of the human genome reveals distinct chromatin-mediated mechanisms for regulating early- and late-firing origins. Genome Res 23, 1774-1788 (2013).

5. Langley AR, Graf S, Smith JC, Krude T. Genome-wide identification and characterisation of human DNA replication origins by initiation site sequencing (ini-seq). Nucleic Acids Res 44, 10230-10247 (2016).

6. Khan A, Mathelier A. Intervene: a tool for intersection and visualization of multiple gene or genomic region sets. BMC Bioinformatics 18, 287 (2017)

7. Marsico G, et al. Whole genome experimental maps of DNA G-quadruplexes in multiple species. Nucleic Acids Res 47, 3862-3874 (2019).

8. Ernst J, Kellis M. ChromHMM: automating chromatin-state discovery and characterization. Nat Methods 9, 215-216 (2012).

9. Natsume T, Kiyomitsu T, Saga Y, Kanemaki MT. Rapid Protein Depletion in Human Cells by Auxin-Inducible Degron Tagging with Short Homology Donors. Cell Rep 15, 210-218 (2016).

10. Yesbolatova A, et al. The auxin-inducible degron 2 technology provides sharp degradation control in yeast, mammalian cells, and mice. Nat Commun 11, 5701 (2020)
