# Supplementary material for: Global landscape of replicative DNA polymerase usage in the human genome
Source: Nat Commun. 2022 Nov 24;13:7221. doi: 10.1038/s41467-022-34929-8 (PMC9700718; doi:10.1038/s41467-022-34929-8)
Supplement: Supplementary file 3 — Reporting Summary [file 41467_2022_34929_MOESM3_ESM.pdf]

nature portfolio

Corresponding author(s): Yasukazu Daigaku

Last updated by author(s): Nov 8, 2022

Reporting Summary

Nature Portfolio wishes to improve the reproducibility of the work that we publish. This form provides structure for consistency and transparency in reporting. For further information on Nature Portfolio policies, see our Editorial Policies and the Editorial Policy Checklist.

Statistics

For all statistical analyses, confirm that the following items are present in the figure legend, table legend, main text, or Methods section.

n/a	Confirmed	
☐	☒	The exact sample size (*n*) for each experimental group/condition, given as a discrete number and unit of measurement
☐	☒	A statement on whether measurements were taken from distinct samples or whether the same sample was measured repeatedly
☐	☒	The statistical test(s) used AND whether they are one- or two-sided<br>*Only common tests should be described solely by name; describe more complex techniques in the Methods section.*
☐	☒	A description of all covariates tested
☐	☒	A description of any assumptions or corrections, such as tests of normality and adjustment for multiple comparisons
☐	☒	A full description of the statistical parameters including central tendency (e.g. means) or other basic estimates (e.g. regression coefficient) AND variation (e.g. standard deviation) or associated estimates of uncertainty (e.g. confidence intervals)
☐	☒	For null hypothesis testing, the test statistic (e.g. *F*, *t*, *r*) with confidence intervals, effect sizes, degrees of freedom and *P* value noted<br>*Give P values as exact values whenever suitable.*
☒	☐	For Bayesian analysis, information on the choice of priors and Markov chain Monte Carlo settings
☒	☐	For hierarchical and complex designs, identification of the appropriate level for tests and full reporting of outcomes
☐	☒	Estimates of effect sizes (e.g. Cohen's *d*, Pearson's *r*), indicating how they were calculated

Our web collection on statistics for biologists contains articles on many of the points above.

Software and code

Policy information about availability of computer code

Data collection	Illumina Hiseq X platform (Illumina), Agilent 4150/4200 TapeStation System (Agilent), ChemiDog Touch (Biorad)
Data analysis	Bowtie2 v2.3.5(http://bowtie-bio.sourceforge.net/bowtie2/manual.shtml)<br>Cufflinks v2.2.1 (http://cole-trapnell-lab.github.io/cufflinks/)<br>STAR v2.7.3a (https://github.com/alexdobin/STAR)<br>Perl script: sam-dup-align-exclude-v2.pl v2.0 (https://github.com/yasukasu/sam-dup-align-exclude).<br>Perl script: pe-sam-to-bincount.pl v1.06 (https://github.com/yasukasu/sam-to-bincount)<br>R script: bincount-csv_to_pol-usage-wig.R v1.0 (https://github.com/yasukasu/Human_Pu-seq)<br>R script: pol-usage-wig_to_ini-index-wig.R v1.0 (https://github.com/yasukasu/Human_Pu-seq)<br>R script: pol-usage-wig_to_fork-index-wig.R v1.0 (https://github.com/yasukasu/Human_Pu-seq)<br>R script: pol-usage-wig_to_coupling-index-wig.R v1.0 (https://github.com/yasukasu/Human_Pu-seq)<br>R language (v4.2.1) for data analysis and graphic visualisation.<br>TapeStation Software v4.1.1 (Agilent)<br>Image Lab v6.1.0(Biorad)

For manuscripts utilizing custom algorithms or software that are central to the research but not yet described in published literature, software must be made available to editors and reviewers. We strongly encourage code deposition in a community repository (e.g. GitHub). See the Nature Portfolio guidelines for submitting code & software for further information.

Data

Policy information about availability of data

All manuscripts must include a data availability statement. This statement should provide the following information, where applicable:

- Accession codes, unique identifiers, or web links for publicly available datasets
- A description of any restrictions on data availability
- For clinical datasets or third party data, please ensure that the statement adheres to our policy

Sequence read and processed data generated in this studies are available in NCBI GEO. Accession ID is GSE189668.
High resolution RT data in HCT116 are available from GSE137764.
OK-seq sequencing read data used in this study are available from NCBI SRA (SRP065949 and SRP144505).
Data for genome-wide G4-duplex formation are available from GSE110582.
ChIP-seq data for histone modification in HCT116 are available from GSE58638.
End-seq data in HCT116 are available from GSE116321
Other source data are provided in a Source Data file.

Human research participants

Policy information about studies involving human research participants and Sex and Gender in Research.

Reporting on sex and gender	N/A
Population characteristics	N/A
Recruitment	N/A
Ethics oversight	N/A

Note that full information on the approval of the study protocol must also be provided in the manuscript.

Field-specific reporting

Please select the one below that is the best fit for your research. If you are not sure, read the appropriate sections before making your selection.

☒ Life sciences ☐ Behavioural & social sciences ☐ Ecological, evolutionary & environmental sciences

For a reference copy of the document with all sections, see nature.com/documents/nr-reporting-summary-flat.pdf

Life sciences study design

All studies must disclose on these points even when the disclosure is negative.

Sample size	Based on the expense of the experiment, three independent pu-seq experiments were performed. In all these experiments, genome-wide profiles of polymerase usage and other processed data were reproducible and thus this sample size was sufficient.
Data exclusions	No data was excluded.
Replication	Information on the number of replicates, samples and independent experiments that were performed for each measurement are disclosed in the manuscript. We replicated our pu-seq experiment in HCT116 cells three times with reproducible outcome. All biological experiments were successfully reproduced in n = 2 or n = 3 independent experiments.
Randomization	No experiment reported were subjected to requirements to ensure a homogeneous population and thus we did not randomize.
Blinding	No experiment reported were subjected to observer bias and thus we did not blind experiments.

Reporting for specific materials, systems and methods

We require information from authors about some types of materials, experimental systems and methods used in many studies. Here, indicate whether each material, system or method listed is relevant to your study. If you are not sure if a list item applies to your research, read the appropriate section before selecting a response.

Materials & experimental systems

n/a	Involved in the study
☐	☒ Antibodies
☐	☒ Eukaryotic cell lines
☒	☐ Palaeontology and archaeology
☒	☐ Animals and other organisms
☒	☐ Clinical data
☒	☐ Dual use research of concern

Methods

n/a	Involved in the study
☒	☐ ChIP-seq
☒	☐ Flow cytometry
☒	☐ MRI-based neuroimaging

Antibodies

Antibodies used	For protein detection, the following commercially available antibodies were used: anti-RNASEH2A (Bethyl Laboratories #A304-149A), anti-alpha-Tubulin (Sigma-Aldrich #T5168), anti-phospho-Chk1 (Cell Signaling Technology #2348), secondary antibodies: anti-rabbit IgG HRP (DAKO P044801-2) and anti-mouse IgG HRP (DAKO, P026002-2).<br>For nucleic acid detection, the following commercially available antibodies were used: anti-BrdU (BD Biosciences 347580) , anti-DNA/RNA hybrid (S9.6) antibody (abcam ab234957) anti-dsDNA antibody (abcam ab27156) and the same secondary antibody as those for protein detection.
Validation	All used primary antibodies were validated by the manufactures and previous publications. References can be found at the manufactures' web site.<br>anti-RNASEH2A<br>https://www.thermofisher.com/antibody/product/RNASEH2A-Antibody-Polyclonal/A304-149A<br>anti-alpha-Tubulin<br>https://www.sigmaaldrich.com/JP/en/product/sigma/t5168?context=product<br>anti-phospho-Chk1<br>https://en.cellsignal.jp/products/primary-antibodies/phospho-chk1-ser345-133d3-rabbit-mab/2348<br>anti-BrdU<br>https://www.bdbiosciences.com/en-eu/products/reagents/flow-cytometry-reagents/clinical-discovery-research/single-color-antibodies-ruo-gmp/purified-mouse-anti-brdu.347580<br>anti-DNA/RNA hybrid (S9.6) antibody<br>https://www.abcam.com/dnarna-hybrid-antibody-s96-ab234957.html<br>anti-dsDNA antibody<br>https://www.abcam.com/ds-dna-antibody-35i9-dna-bsa-and-azide-free-ab27156.html

Eukaryotic cell lines

Policy information about cell lines and Sex and Gender in Research

Cell line source(s)	HCT116 line was originally obtained from ATCC .
Authentication	The original HCT116 were authenticated by ATCC. Other HCT116 derivative cell lines were not authenticated. However, all HCT116 cell lines used in this study were frequently checked their morphology, growth, and karyotype.
Mycoplasma contamination	All cell lined used is negative in Mycoplasma contamination test by PCR prior to experiments.
Commonly misidentified lines (See ICLAC register)	No commonly misidentified cell lines were used.
